# Pest population dynamics are related to a continental overwintering gradient

**DOI:** 10.1073/pnas.2203230119

**Published:** 2022-09-06

**Authors:** Douglas Lawton, Anders S. Huseth, George G. Kennedy, Amy C. Morey, William D. Hutchison, Dominic D. Reisig, Seth J. Dorman, DeShae Dillard, Robert C. Venette, Russell L. Groves, John J. Adamczyk, Izailda Barbosa Dos Santos, Tracey Baute, Sebe Brown, Eric Burkness, Ashley Dean, Galen P. Dively, Hélène B. Doughty, Shelby J. Fleischer, Jessica Green, Jeremy K. Greene, Krista Hamilton, Erin Hodgson, Thomas Hunt, David Kerns, Billy Rogers Leonard, Sean Malone, Fred Musser, David Owens, John C. Palumbo, Silvana Paula-Moraes, Julie A. Peterson, Ricardo Ramirez, Silvia I. Rondon, Tracy L. Schilder, Abby Seaman, Lori Spears, Scott D. Stewart, Sally Taylor, Tyler Towles, Celeste Welty, Joanne Whalen, Robert Wright, Marion Zuefle

**Affiliations:** ^a^Department of Entomology and Plant Pathology, North Carolina State University, Raleigh, NC 27695;; ^b^North Carolina Plant Sciences Initiative, North Carolina State University, Raleigh, NC 27606;; ^c^Department of Entomology, University of Minnesota, St. Paul, MN 55108;; ^d^Forage Seed and Cereal Research Unit, Agricultural Research Service, US Department of Agriculture (USDA), Corvallis, OR 97331;; ^e^Northern Research Station, USDA Forest Service, St. Paul, MN 55108;; ^f^Department of Entomology, University of Wisconsin, Madison, WI 53706;; ^g^Thad Cochran Southern Horticultural Laboratory, USDA-Agricultural Research Service, Poplarville, MS 39470;; ^h^West Florida Research and Education Center, University of Florida, Jay, FL 32565;; ^i^Great Lakes and Maritimes Pest Monitoring Network, Food and Rural Affairs, Ontario Ministry of Agriculture, Ridgetown, ON, N0P 2C0, Canada;; ^j^Department of Entomology and Plant Pathology, University of Tennessee, Jackson, TN 38301;; ^k^Department of Entomology, Iowa State University, Ames, IA 50011;; ^l^Department of Entomology, University of Maryland, College Park, MD 20742;; ^m^Eastern Shore Agricultural Research and Extension Center, Virginia Tech, Painter, VA 23420;; ^n^Department of Entomology, Pennsylvania State University, University Park, PA 16802;; ^o^Department of Horticulture, Oregon State University, Corvallis, OR 97331;; ^p^Department of Plant and Environmental Sciences, Clemson University, Blackville, SC 29817;; ^q^Pest Survey Program, Wisconsin Department of Agriculture, Trade and Consumer Protection, La Crosse, WI 54601;; ^r^Department of Entomology, University of Nebraska–Lincoln, Lincoln, NE 68588;; ^s^Department of Entomology, Texas A&M University, College Station, TX 77843;; ^t^Department of Entomology, Louisiana State University Agricultural Center, Baton Rouge, LA 70803;; ^u^Department of Entomology, Virginia Tech, Suffolk, VA 23437;; ^v^Department of Biochemistry, Molecular Biology, Entomology, and Plant Pathology, Mississippi State University, Mississippi State, MS 39762;; ^w^Carvel Research and Education Center, University of Delaware, Georgetown, DE 19947;; ^x^Yuma Agricultural Center, University of Arizona, Yuma, AZ 85364;; ^y^Department of Entomology, University of Nebraska–Lincoln, North Platte, NE 69101;; ^z^Department of Biology, Utah State University, Logan, UT 84321;; ^aa^Oregon Integrated Pest Management (IPM) Center, College of Agricultural Sciences, Oregon State University, Corvallis, OR 97333;; ^bb^Hermiston Agricultural Research and Extension Center, College of Agricultural Sciences, Oregon State University, Corvallis, OR 97838;; ^cc^New York State IPM Program, College of Agriculture and Life Sciences, Cornell University, Geneva, NY 14456;; ^dd^Pest Survey Program, Wisconsin Department of Agriculture, Trade and Consumer Protection, Madison, WI 53718;; ^ee^Macon Ridge Research Station, Louisiana State University, Winnsboro, LA 71295;; ^ff^Department of Entomology, Ohio State University, Columbus, OH 43210;; ^gg^Department of Entomology and Wildlife Ecology, University of Delaware, Newark, DE 19716

**Keywords:** bollworm, corn earworm, dispersal, long-term monitoring, migration

## Abstract

Overwintering success is an important determinant of arthropod populations that must be considered as climate change continues to influence the spatiotemporal population dynamics of agricultural pests. Using a long-term monitoring database and biologically relevant overwintering zones, we modeled the annual and seasonal population dynamics of a common pest, *Helicoverpa zea* (Boddie), based on three overwintering suitability zones throughout North America using four decades of soil temperatures: the southern range (able to persist through winter), transitional zone (uncertain overwintering survivorship), and northern limits (unable to survive winter). Our model indicates *H. zea* population dynamics are hierarchically structured with continental-level effects that are partitioned into three geographic zones. Seasonal populations were initially detected in the southern range, where they experienced multiple large population peaks. All three zones experienced a final peak between late July (southern range) and mid-August to mid-September (transitional zone and northern limits). The southern range expanded by 3% since 1981 and is projected to increase by twofold by 2099 but the areas of other zones are expected to decrease in the future. These changes suggest larger populations may persist at higher latitudes in the future due to reduced low-temperature lethal events during winter. Because *H. zea* is a highly migratory pest, predicting when populations accumulate in one region can inform synchronous or lagged population development in other regions. We show the value of combining long-term datasets, remotely sensed data, and laboratory findings to inform forecasting of insect pests.

## Significance

The expansion of pest ranges due to climate change will threaten global agriculture. Winter soil temperature is known to limit pest persistence at higher latitudes. However, few studies have connected overwintering success of soil-dwelling insects with long-term population datasets to investigate how climate change may affect pests’ distributions and population dynamics in the future. Here, we present models demonstrating how greater overwintering survival may expand the range of a serious insect pest. We also highlight the need for projected soil temperature data based on climate change scenarios. To ensure sustainable agricultural production, it is imperative that insect pest range shifts are anticipated to develop solutions that mitigate crop loss in expansion areas.

Animal population dynamics often reflect large-scale climatic variations (1). For example, many insects have short annual cycles, with elevated population densities during warm summer months (2, 3), while populations of other animals respond to long-term climatic oscillations (4, 5). Thus, understanding how animal population dynamics are influenced by climatic conditions has been an important research theme in modern ecology.

Understanding the seasonality of pest population dynamics is essential to implementing integrated pest management (IPM) strategies to reduce dependence on pesticide applications and preserve ecosystem services while ensuring food security (6, 7). In this context, it is important to understand how long-term trends in pest population dynamics are predicted by climatic seasonality as climate change alters species ranges (8). For many insects, winter temperatures are one of the fundamental abiotic factors limiting range expansion into higher latitudes or elevations. With climate change, warmer winters may increase the land area suitable for insect overwintering, which, in turn, is predicted to increase crop damage and pesticide use and resistance in some species (9, 10). Therefore, it is important to document baseline trends to track annual or interannual climate change effects on pests prior to the expansion of overwintering ranges. Here, we investigate the impact of a changing climate on seasonal population dynamics of corn earworm, *Helicoverpa zea* (Boddie, 1850), in North America by using long-term monitoring datasets, remotely sensed weather data, and laboratory findings.

*H. zea* is an excellent study organism for observing gradual effects of climatic seasonality on pest population dynamics because its geographic distribution spans a broad temperature gradient in North America. It is a polyphagous, highly migratory, and multivoltine lepidopteran pest of crops such as maize, cotton, soybeans, and vegetables, and feeds on many noncrop hosts (11–13). *H. zea* pupae undergo a facultative winter diapause that enables them to overwinter underground below the 40°N latitude in North America (14, 15). It is generally accepted that few individuals overwinter above this 40°N latitude due to lethal winter temperatures (14). However, its summer range expands north to approximately the 52°N latitude (14). Seasonal *H. zea* populations have been monitored across broad regions of North America over decades to estimate their activity and potential for crop infestation (16). This long-term dataset provides opportunity to gain insight into the relationship between climate, seasonal population dynamics, and range expansion.

Prior efforts to model *H. zea* overwintering biology that relate laboratory estimates of low-temperature tolerance thresholds to climate data have agreed with historical reports that successful overwintering is limited to latitudes below 40°N latitude (17). However, there have been no reported models directly linking *H. zea* observational data with remotely sensed data to determine the relationship between climatic seasonality and the continental-scale population dynamics of this important pest species. Remotely sensed data provide measurements of environmental processes that enable large-scale modeling efforts not previously possible. Connecting temperature-mediated overwintering zones to long-term *H. zea* population datasets and remotely sensed data can provide a foundation to monitor and project population changes under current and future climate change scenarios.

Evidence supports that in-season temperature differences directly affect *H. zea* population dynamics in conjunction with host phenology and cropping patterns and that they do so differently at a regional scale (18). We know populations migrate northward in a somewhat predictable way. Using a combination of radar and pollen identification, researchers have confirmed that spring *H. zea* populations undergo long-distance dispersal each year (19–21). There is abundant evidence that *H. zea* does not effectively overwinter in the more northerly regions, where it is a later-season pest in most years (14, 22). Based on this, we expect the timing and severity of problems in northern regions to be influenced by the southern *H. zea* populations that develop and are sources of migrants. Other within-season factors can also affect regional population dynamics. These include but are not limited to cropping patterns and practices, host phenology, spring and summer temperatures, precipitation, and pest management interventions (23–26). We also expect overwintering survival, especially in marginal areas, to vary depending on winter temperatures. This variation may affect intra- and interregional dynamics by altering timing and magnitude of population development and the role of migration from core overwintering areas. On this basis, we investigated the relationship between predicted *H. zea* overwintering success based on soil temperatures and continental-scale variation in its population dynamics.

Because winter soil temperature is a critical determinate of overwintering survival (27), we investigated the relationship between overwintering conditions and historical *H. zea* population dynamics. We show how changes in climate may translate into a projected shift in the overwintering range of *H. zea*. To do this, we integrated long-term *H. zea* adult moth abundance datasets with laboratory data on low-temperature survival thresholds for diapausing pupae and remotely sensed temperature data. We used these data to identify three broad zones throughout North America representing likely overwintering survival based on soil temperature suitability. These zones are: the southern range, where overwintering success is predictable; transitional zone, where overwintering success is expected to vary based on winter temperatures; and northern limits, where lethal winter temperatures are expected to prevent survival. We then constructed generalized additive mixed models (GAMMs) with varying hierarchical structure (no structure to global- and group-level structure) connecting *H. zea* population dynamics to the overwintering zones.

**Fig. 1.**
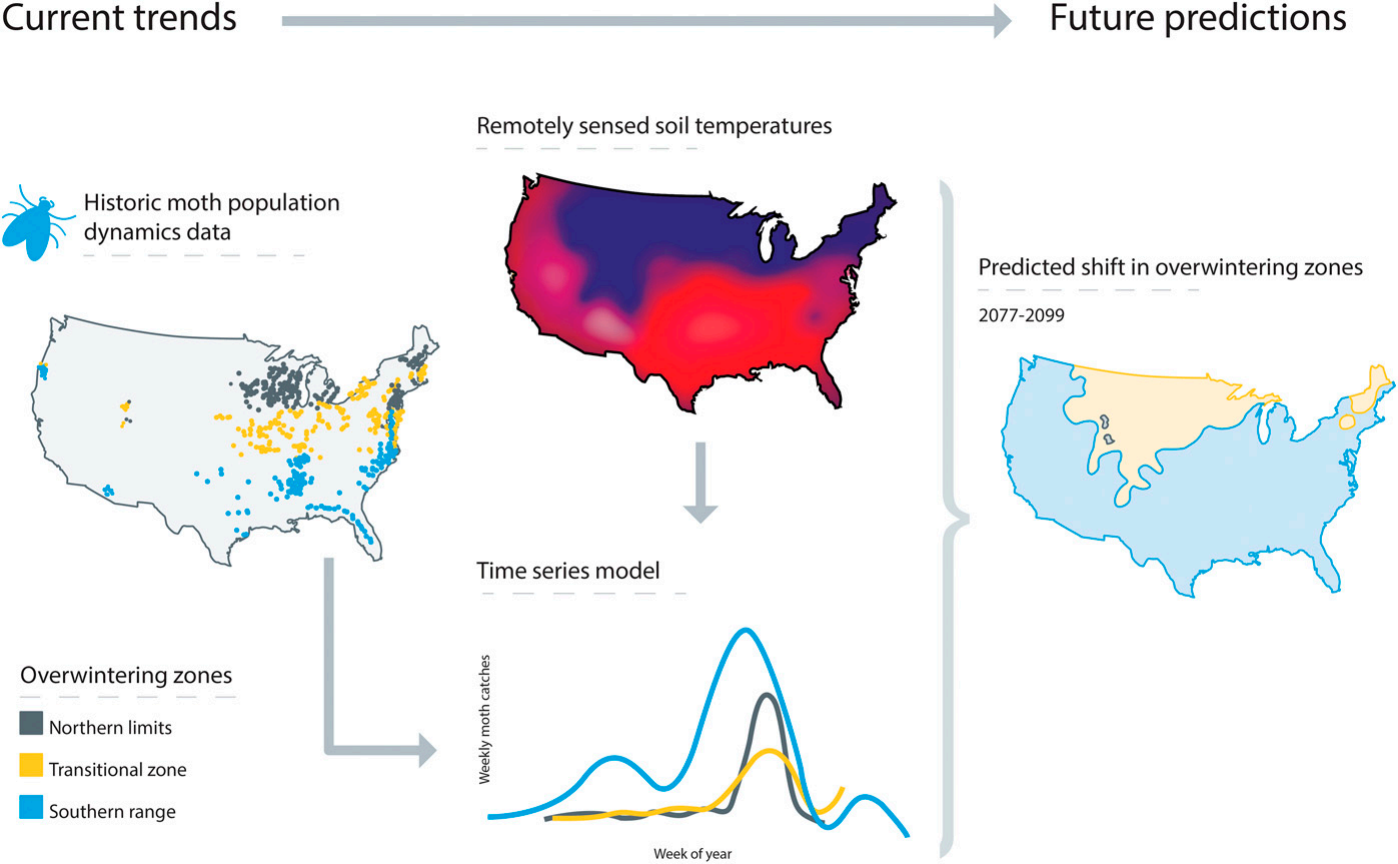
Overview of our modeling approach. We constructed overwintering zones based on laboratory cold tolerance studies with remotely sensed climatic reanalysis data. Then, we partitioned *H. zea* population dynamics based on the overwintering zones. Lastly, we projected the *H. zea* overwintering range into the future by relating current soil and air temperature data and predicting future soil temperature using future air temperature conditions.

We hypothesized that *H. zea* population dynamics will vary based on broad zones of soil temperature suitability. Specifically: Populations in the southern range, where overwintering success is predictable, will increase and reach higher levels earlier in the season compared with both the transitional zone, where overwintering success is uncertain, and the northern limits, where lethal winter temperatures are expected to prevent survival. Because *H. zea* is migratory, we expected to see a signal of seasonal northward migration in the form of a predictable increase in northern populations following peak abundance in the south. Lastly, we forecast how the three overwintering zones may change in area based on climate change scenarios. Our results highlight the potential for range expansion and changes in the seasonal abundance of this important crop pest due to increasing temperature suitability in northern latitudes as the climate changes. We visually outline our approach in Fig. 1.

## Results

### Overwintering Zone Classification.

**Fig. 2.**
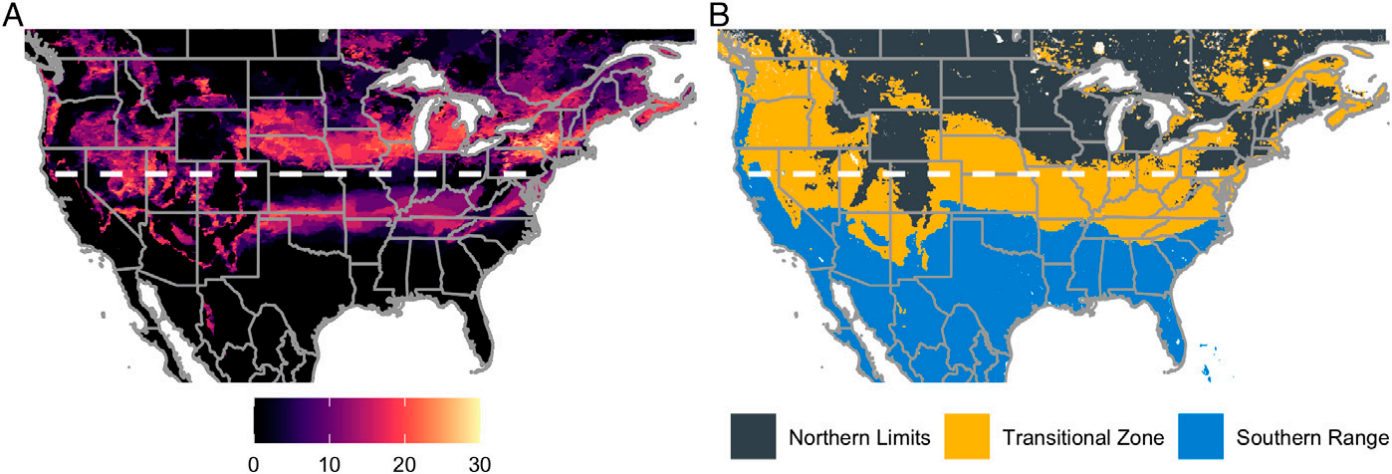
Overwintering zone classification based on a 40-y averaged modeled soil temperature (0 to 28 cm). (*A*) How often year-to-year changes occur between the three overwintering zones. The scale represents the number of between-year changes between the three zones. For example, if a pixel switched between the northern limits and transitional zone between the years 2001 and 2002, we assigned a value of 1 and summed up all between-year changes for the available data. (*B*) The 40-y averaged zone classification. Grey dashed lines indicate the location of the widely accepted northern overwintering limit of the 40°N latitude.

We examined the widely accepted northern overwintering limit of the 40°N latitude and found that there is significant spatial and temporal variation around this line (Fig. 2*A*). A 40-y averaged overwintering map constructed based on remotely sensed soil temperature projected the transitional zone to be largest in area, followed by the southern range and then the northern limits (Fig. 2*B*). Over the past 40 y, changes in temperature predict a consistent expansion of the southern range and transitional zone and a decrease in the northern limits after 2000 (*SI Appendix*, Fig. S1). These changes are visualized in Movie S1.

We tested the validity of our overwintering zones using the Akaike information criterion (AIC) and Bayesian information criterion (BIC). Both overwhelmingly selected a model partitioning of 40-y averaged overwintering into three zones as compared with other models tested including the 40°N latitude (*SI Appendix*, Table S1). This result indicates the importance of a transitional zone to reflect winter soil temperature uncertainty between the overwintering southern areas and the lethal northern areas and suggests a temporal lag effect of winter soil temperatures on *H. zea* population dynamics.

Using the three overwintering zone partitions in the final hierarchical models, we then show that *H. zea* population dynamics are structured with a global and three, similarly smoothed, overwintering zone group levels (model GS; *SI Appendix*, Table S2). Acknowledging this hierarchy improved model fit, increasing adjusted *R*-squared values from 0.13 (model N, a model with no structure) to 0.30 (model GS; *SI Appendix*, Table S3). Model predictions were more certain about population dynamics in the northern limits and southern range than the transitional zone (*SI Appendix*, Table S4). This hierarchical model (*SI Appendix*, Table S2) had the best or second-best predictive ability for both the northern limits and transitional zone but not the southern range, where a model (model S; *SI Appendix*, Table S5) with similarly smoothed group levels only was the best predictor. *H. zea* sample distributions and all other model summaries are reported in *SI Appendix*, Figs. S2 and S3 and Tables S6 and S7. A latitude–longitude–year tensor and spatial correlation of remaining residuals are shown in *SI Appendix*, Figs. S4 and S5.

### *H. zea* Population Dynamics.

**Fig. 3.**
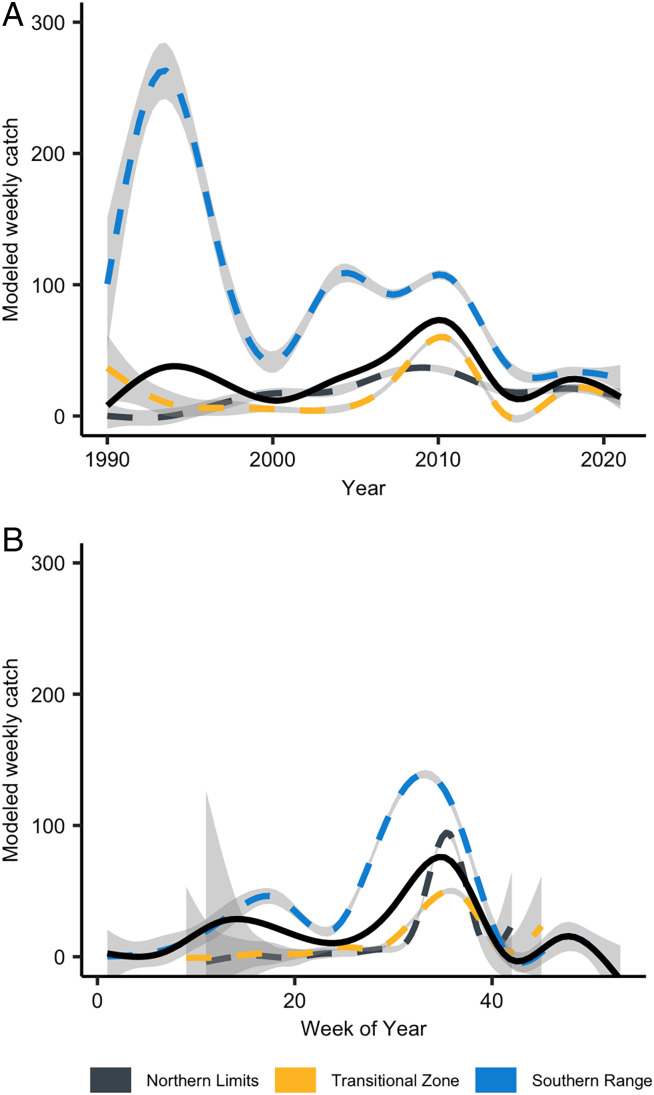
*H. zea* population dynamics among (*A*) and within (*B*) years. Figures are final model predictions (model GS) for year and week of year, respectively. Solid black lines represent the global (or species range) and dashed lines represent the population dynamics within the overwintering zones. Gray shaded areas represent model uncertainty.

*H. zea* populations displayed nonlinear trends between and within years (Fig. 3 and *SI Appendix*, Table S2). Overall, three clear interannual population peaks occurred in 1995, 2004, and around 2010 (Fig. 3*A*). The southern range had more dynamic population booms and busts when compared with the other two zones and the populations in the transitional zone were more dynamic than in the northern limits. Averaged across zones, intraannual population dynamics were characterized with three peaks roughly at weeks 18, 35, and 42 (Fig. 3*B*). This global trend is divided into the overwintering zones, with only the southern range model fits exhibiting early and midseason peaks around week of year 18 and 32, respectively (Fig. 3*B*). The northern limits and transitional zone models exhibited one midseason peak around week 35, which aligns with a northward temporal lag across the zones, with the southern range peaking first followed by peaks in the transitional zone and northern limits 4 to 5 wk later. Southern range annual population peaks were highly positively correlated with the transitional zone (rho = 0.75; *SI Appendix*, Fig. S6*A*) and northern limits peaks (rho = 0.49; *SI Appendix*, Fig. S6*B*). And transitional zone yearly peaks were also highly correlated with northern limits yearly peaks (rho = 0.81; *SI Appendix*, Fig. S6*C*). Southern range populations were higher throughout the year on average than in the other regions.

### Future Change of Overwintering Zones.

**Fig. 4.**
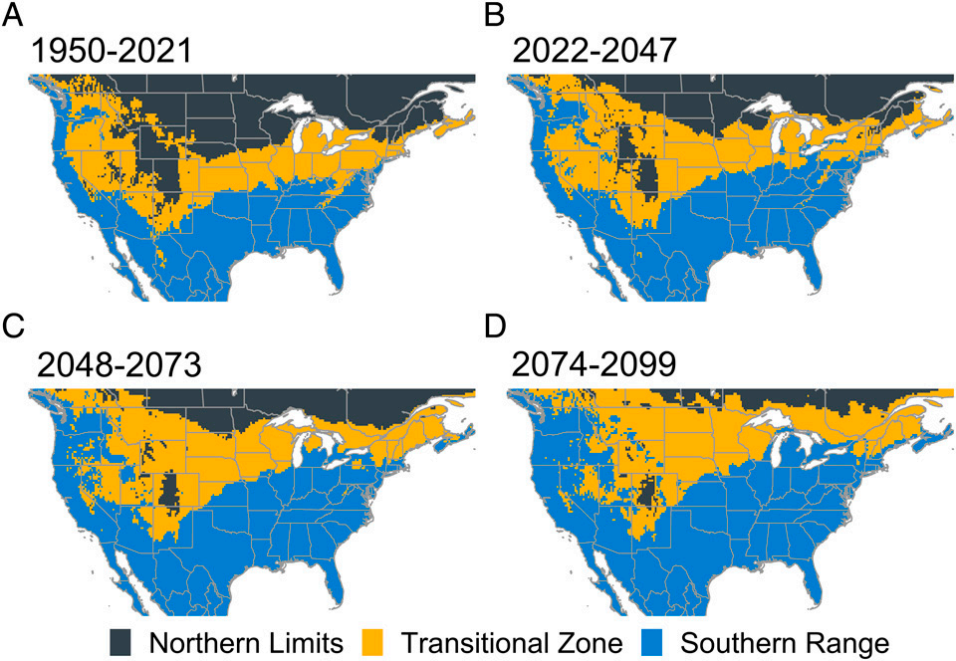
Projected overwintering zone change from historic and current averages to 2099. Area was estimated using NASA’s Earth Exchange Global Daily Downscaled Climate Projection under RCP 8.5 adjusted to soil temperatures. Because these data were derived from projected air temperatures, they only provide a coarse understanding of potential overwintering zone shift.

To forecast changes in overwintering zone areas in response to climate change, we first predicted soil temperature from future air temperatures until 2099 using a simple GAM (adjusted *R*-squared: 0.87) modeling the relationship between air and soil temperature (*SI Appendix*, Fig. S7). Then, we used this relationship to visualize forecasted changes in overwintering zones based on projected soil temperature (Fig. 4). These forecasts predict an expansion of the southern range from 34 to 56% and a major decrease in the northern limits from 39 to 11% of the proportional area with temperature change into the next century.

## Discussion

Our findings, using one of the largest spatiotemporal datasets compiled for a migratory agricultural pest in North America, are consistent with multiscale climatic seasonality of *H. zea* population dynamics at scales ranging from continental to three distinct zones across North America that are expected to differ in overwintering success. Here, we linked long-term observational data on *H. zea* adult activity to overwintering zones defined by remotely sensed soil temperature and laboratory survival data. Our spatially explicit approach refines the historically proposed 40°N latitude limit for overwintering populations (14, 15), shifting it to 35°N latitude in the southwestern United States, a distance of ∼555 km (Fig. 2*B*). This suggests a need to reassess this overwintering zone division, as it is a common spatial divide used for established and potentially invasive insect pest species. Our models describe temporally lagged associations across zones of overwintering success between winter soil temperature and *H. zea* population dynamics, consistent with migration from southern areas acting as a driver of dynamics north of their overwintering range. This result agrees with studies focused on northward *H. zea* migration in North America (19–21). The specific areas of these zones are predicted to change based on climatic fluctuation, both in terms of large-scale climatic patterns (e.g., sea surface temperature anomalies) and anthropogenically induced climate change. Our results suggest that the spatial extent of overwintering suitability has been changing over the past decade with an expansion of southern range area (*SI Appendix*, Fig. S1).

A surprising outcome of this work was the selection of the 40-y average as the most meaningful predictor of *H. zea* population dynamics. If overwintering mortality is the key factor driving population dynamics, we would expect conditions within the preceding winter to be most important. Logically, these effects should be strongest in areas where the frequency of change between suitable and unsuitable diapause conditions is high, specifically at the interface between the transitional zone and northern limits (Fig. 2*A*). However, it is reasonable to expect that other factors beyond overwintering mortality also play a role governing *H. zea* populations. One clear example is the role of host crop availability in agricultural landscapes, which can affect the abundance of this pest. The effects of annual cropping cycles are strongest in the southern United States, where a sequence of suitable *H. zea* hosts are grown each year (23–25, 28). As a result, fluctuations in the abundance and composition of host crop acreage over time may be another explanation for the limited effect of annual winter temperatures.

Our analyses of moth-trapping data revealed *H. zea* population dynamics are hierarchically structured with overall and nested effects between the overwintering zones. Such changes in population patterns based on spatial scale are common in ecology (29). Without acknowledging this, researchers and practitioners risk erroneous conclusions about population dynamics among spatial scales (30). *H. zea* are highly migratory, capable of dispersing at least 600 to 1,000 km by using seasonal wind patterns (19, 31). Thus, population dynamics of one region likely influence the dynamics in distant regions, with practical consequences for pest management targeting *H. zea*. Hence, accounting for migration is critical in predicting when and where *H. zea* populations proliferate at the continental scale.

Our analysis showed that, except for 2010, when all regions experienced an uptick in *H. zea* densities, population dynamics were not synchronized between regions interannually. This suggests that regional abiotic and biotic factors not included in our models likely explain the limited within-region descriptive capability of our coarse models. These differences may have resulted from changes in the agricultural production system, abiotic conditions, and/or landscape composition. For example, the natural population cycles among years could have been disrupted by adoption of *Bacillus thuringiensis* (*Bt*) maize and cotton starting in 1996 (26). Although we did not have sufficient historical crop production data to illustrate the disruptive effect of *Bt* adoption on *H. zea*, *Bt* crops are known to reduce the probability of crop injury by both *H. zea* (26) and *Helicoverpa armigera*, a cosmopolitan pest species in the same genus that fills a similar niche (32). Although the *Bt* crops have been shown to suppress polyphagous lepidopteran pests, the longevity of this benefit is a significant concern. Recent, widespread evolution of *Bt* resistance in *H. zea* populations, now common in all overwintering zones, may alter the continental-scale population dynamics and undermine the benefit of damage suppression in the eastern United States (27, 28, 33–35). Clearly, annual changes in population dynamics reported in this study must be accounted for to understand the long-term effects of widespread *Bt* adoption on *H. zea*.

Land management practices are constantly changing, impacting overall landscape suitability for many insect pest species (36–38). The abundance of *H. zea* habitat within overwintering zones varies greatly, and both regional and annual variability in abundance of key host crops (maize, soybean, and cotton) may explain additional noise in our dataset (39).

Within a given year, our results support the importance of knowing when southern range populations of *H. zea* build and adults migrate into the two northern regions (40). Our simple interannual peak correlations predict temporally lagged, strong positive associations between overwintering zones (rho = 0.49 to 0.81; *SI Appendix*, Fig. S6), which would enable predictions of when *H. zea* migrants are expected to reach the transitional zone and northern limits, given the timing of population development in the southern range. Interregion monitoring efforts could improve smaller-scale tracking efforts within individual *H. zea* regions (16). For a migratory species like *H. zea*, effective monitoring at the continental scale is necessary to have the greatest management effect (41). This requires a coordinated network of stakeholders, from growers to extension agents and crop advisors, actively communicating among crop regions.

With climate change, seasonal patterns are changing. As a result, *H. zea* and other animal overwintering distributions are being influenced (42). While there is limited understanding of future soil temperatures, our coarse predictions of future *H. zea* overwintering zones (Fig. 3) suggest that the historical drivers of population abundance and distribution will likely change with warming winter temperatures. An important aspect of the transitional zone’s forecasted northward shift (Fig. 4) is increased year-to-year uncertainty in overwintering success and, consequently, the potential for damaging *H. zea* populations. Successful overwintering of *H. zea* populations at higher latitudes due to warmer winters can be expected to affect the timing and intensity of populations in regions currently only accessible to migrants that arrive later in the season. Historically, the US maize belt has been too cold for this species to overwinter (14, 27). However, warmer winter temperatures expected in the future may allow early-season population increases resulting from increased overwintering success and annual migration from the southern range (Fig. 3) that may result in increased pesticide use and yield loss in northern maize production regions. Even though some regions experience frequent zone changes throughout the years (Fig. 2*A*), our model selection criteria selected for the 40-y averaged zone as compared with the year-to-year overwintering zones. This suggests that there are temporally lagged associations between yearly zone classifications at this level. However, it is reasonable to expect that year-to-year zone change may be important, and this merits further investigation.

In interpreting our results, it is important to note that we used a simple definition of overwintering success. We did not directly model overwintering survival; rather, we inferred survival based on seasonal population dynamics. A more comprehensive model including other survival-limiting factors such as lethal event duration (e.g., time spent below 0 °C) would increase model accuracy. Whereas the relationship between overwintering temperatures and insect overwintering success has been shown in other insects (17, 42–44), the ability to forecast distributions of species that overwinter in buffered microhabitats such as soil is limited due to lack of available relevant climate data. We used a simplified relationship between air and soil temperature to forecast the impact of climate change on our overwintering zones, and careful interpretation is needed for our projected overwintering zones. Further development of global, high-resolution datasets is needed to predict species distributions for organisms that are limited by winter soil temperature. Our study highlights the need for research modeling of future soil temperatures and their relationship to both host range and population dynamics of agricultural pests, including *H. zea*. Developing biologically relevant models that illuminate how climate change may impact the overwintering success of insects is imperative for sustainable crop protection that alleviates the negative impacts of pesticides on rural communities and the environment.

## Materials and Methods

### Overwintering Zone Classification.

To determine where *H. zea* can overwinter throughout North America, we used experimental thresholds generated by Morey et al. (27) that show diapausing *H. zea* pupae had significantly higher mortality below 0 °C and minimal mortality at 5 °C. Although many variables influence overwintering success (45), including time spent at a given temperature and the interaction with soil moisture, we assume averaged winter soil temperature to be a suitable proxy for complex interactions between soil temperature and diapause success, in that decreasing temperatures imply increasing probability of mortality. We used this cold tolerance survival as a conservative threshold to construct three broad survival zones:

Southern range, where the mean minimum soil temperature in winter is above 5 °C;
Transitional zone, where the soil temperatures range from 0 to 5 °C;
Northern limits, where the soil temperature is below 0 °C.

These zones reflect areas where *H. zea* is likely to overwinter (southern range), potentially overwinter given the specific year (transitional zone), and unlikely to overwinter (northern limits). The transitional zone is a zone of uncertainty between the two major regions that accounts for variation between zones based on winter soil temperatures and other abiotic and biotic factors limiting overwintering survival.

For map construction, we extracted mean minimum winter (December to February) soil temperature to approximate the winter temperature extremes experienced from the Copernicus Climate Change Service’s ERA5-Land climate reanalysis database (46). This database provides hourly estimates of various climatic variables from 1981 to the present at a spatial resolution of 0.1 × 0.1° (native resolution of 9 km). To reflect the variability of where *H. zea* diapause as pupae in the soil profile across the continent and the available soil depths within the climatic reanalysis dataset, we averaged soil temperatures from 0 to 28 cm below the surface (14). We then binned cell values as one of the three zones for each year based on the temperature ranges discussed above.

To visualize interannual changes in the three overwintering zones, we constructed a raster with 41 bands representing the zones from 1981 to 2021 and a 40-y averaged zone map (Fig. 2*B*). We then produced a static map (Fig. 2*A*) and video to illustrate how the area of each zone varies over time (Movie S1).

### *H. zea* Database Construction.

Historical *H. zea* data were retrieved from public and private organizations throughout the United States and Canada (*SI Appendix*, Fig. S2). The data consist of georeferenced periodic adult *H. zea* counts caught in 1,986 unique pheromone and black light traps located in 37 US states and one Canadian province (47). The combined dataset included over 100,000 unique *H. zea* observations from the early 1980s to 2021. Traps were checked either daily or weekly, although some were checked less frequently. To homogenize the structure between all sources, we summed up all counts to the International Organization for Standardization (ISO) 8601 system’s week of year, including a recurring leap week. For traps checked irregularly and for longer than 1 wk, we divided the *H. zea* count by the number of days the trap was operational and multiplied by seven to have an approximate weekly rate. Pheromone trap lures were changed approximately every 2 wk. Trap locations were biased toward the eastern seaboard, where *H. zea* is a persistent pest of multiple crops; however, trap locations from several western states were also included (*SI Appendix*, Fig. S2). We were able to account for differences between pheromone and black light trap catches by including trap location and trap type as a random effect in our analyses. Trapping usually happens during season and therefore a limitation of this dataset is it is unlikely to capture nongrowing season population dynamics. We have provided density plots for *H. zea* count, week of the year, and year (*SI Appendix*, Fig. S3).

### Statistical Analysis.

To evaluate how *H. zea* population dynamics vary between the overwintering zones, we constructed hierarchical GAMMs. These models allow for the relationship between the dependent and independent variables to be smoothed and thus allow nonlinear relationships (48). This flexibility leads to an easy extension into hierarchical modeling where the smoothed relationships vary between groups (49).

To test the overwintering zone structure, we constructed six models: a model with three, 40-y averaged overwintering zones; a model with two 40-y averaged overwintering zones (dissolving the transitional zone into the southern range); two seasonal models with the same structure as the 40-y averaged models; a model that reflected the 40°N latitude split between overwintering and nonoverwintering zones; and a model with no partitioning at all. Each model included the following independent variables: week of year and year as fixed effects, and trap location and their respective zone partitioning as random effects. We selected the best fit model based on AIC and BIC.

We fitted GAMMs to *H. zea* count data with the following variables: week of year, year, longitude/latitude, trap location, trap type, and overwintering zone. These variables were specified differently based on a hierarchical structure, as explained below. All models had a space–time tensor of two-dimensional latitude and longitude smooths and one-dimensional year term (using Gaussian process bases), which models the spatial and yearly temporal component of *H. zea* counts to account for autocorrelation. We used the Tweedie distribution as discussed in *SI Appendix* Text, S1.

To test the hierarchical nature of the overwintering zones, we constructed six models with a varying structure following methods similar to other studies (30, 49). We constructed two models with a global smoother (the entire dataset extent relationship) and group-level trends that were either similarly smoothed (group-level relationships are modeled dependent on each other; model GS) or differently smoothed trends (group-level relationships are modeled independently; model GI); two models without a global smoother but with group-level trends either similarly or differently smoothed (models S and I, respectively); one model with a global-level smoother only (model G); and a model with no hierarchy (model N). This allowed us to test for multiscale *H. zea* population dynamics with two spatial levels: the entire dataset level (e.g., global trend) and three nested overwintering zones (e.g., group-level trends).

All models were verified via diagnostic plots. We ensured basis dimensions were large enough to capture nonlinear trends (*SI Appendix*, Tables S8–S10). We used null space penalization to test the presence of nonlinear trends in the model (48, 50). The final model was chosen using five model selection criteria to account for discrepancies between criteria (51). We calculated the AIC and BIC from models built with all data followed by 10-fold cross-validation using randomly sampled training (70%) and testing (30%) splits of weekly moth counts to test the predictive ability of all models. We calculated out of sample deviance (49) and root-mean-square error between known testing data and model predictive values and averaged all fold model adjusted *R*-squared values together. The final model had the most support from all selection criteria.

We used Pearson correlation of the peak week of year range per zone as determined by the overall GAMM to understand the relationship between *H. zea* peak populations among the overwintering zones. This allowed us to associate the peak abundance week for each zone in each year as a rough estimate of interzone correlations.

To demonstrate potential overwintering zone change driven by future climate change, we constructed future soil temperature maps based on the most extreme representative concentration pathway (RCP 8.5). Due to limited availability of future soil temperature projections, we used a GAM (family: Gaussian; link: identity) with historical soil and air temperature as the dependent and independent variables, to predict future mean minimum winter soil temperature for the years 2022 to 2047, 2048 to 2073, and 2074 to 2099. We used NASA’s Earth Exchange Global Daily Downscaled Climate Projection (52), which is a dataset composed of 22 different climatic scenarios which we averaged with equal weighting together to produce ensemble predictions for historical and future air temperatures and the ERA5 dataset for historical soil temperatures at 0- to 28-cm depth. We assumed a relationship between air and soil temperature (53, 54). However, we acknowledge that several factors can also influence soil temperatures, including snow cover, litter cover, and soil type (55–57). Hence, our approach likely over- and underestimates the overwintering zones in some areas. As such, these maps were generated to provide a visual illustration and represent a coarse understanding of how *H. zea* overwintering zones may change in the future. All software used is discussed in *SI Appendix*.

## Supplementary Material

Supplementary File

Supplementary File

## Data Availability

The dataset and modeling script reported in this article have been deposited in Dryad, https://doi.org/10.5061/dryad.m0cfxpp5x (47).
