# Supplementary material for: Pest population dynamics are related to a continental overwintering gradient
Source: Proc Natl Acad Sci U S A. 2022 Sep 6;119(37):e2203230119. doi: 10.1073/pnas.2203230119 (PMC9477387; doi:10.1073/pnas.2203230119)
Supplement: Supplementary File [file pnas.2203230119.sapp.pdf]

www.pnas.org

Supplementary Information for

Pest population dynamics are driven by a continental overwintering gradient

Lawton et al.

Douglas Lawton and Anders Huseth.
E-mail: ddlawton@ncsu.edu and ashuseth@ncsu.edu

This PDF file includes:

Supplementary text
Figs. S1 to S7
Tables S1 to S10
Legend for Movie S1
SI References

Other supplementary materials for this manuscript include the following:

Movie S1

Supporting Information Text

Supporting Information Text

Tweedie distribution discussion.

To account for overdisperion, we used the Tweedie distribution (link: log) (1), which is a special type of dispersion model where the power (p) value defines the distribution (Gaussian: p = 0; Poisson: p = 1; gamma: p = 2). The power value can be estimated via maximum likelihood during model fitting, and when the value is between zero, one, and two, the model is a mixture that can help with zero-inflated data, much like quasi-distributions (2).

Software used.

All statistics and *H. zea* data management were done in R (3) within the *tidyverse* framework (4). Overwintering zones classification was done in Google Earth Engine (5). Models were built with *mgcv* (2) and validated with *gratia* (6). For data management and vizualization, we used *lubridate* (7), *sp* (8), *sf* (9), *raster* (10), *rnaturalearth* (11), *rgdal* (12), and *SpatialEco* (13).

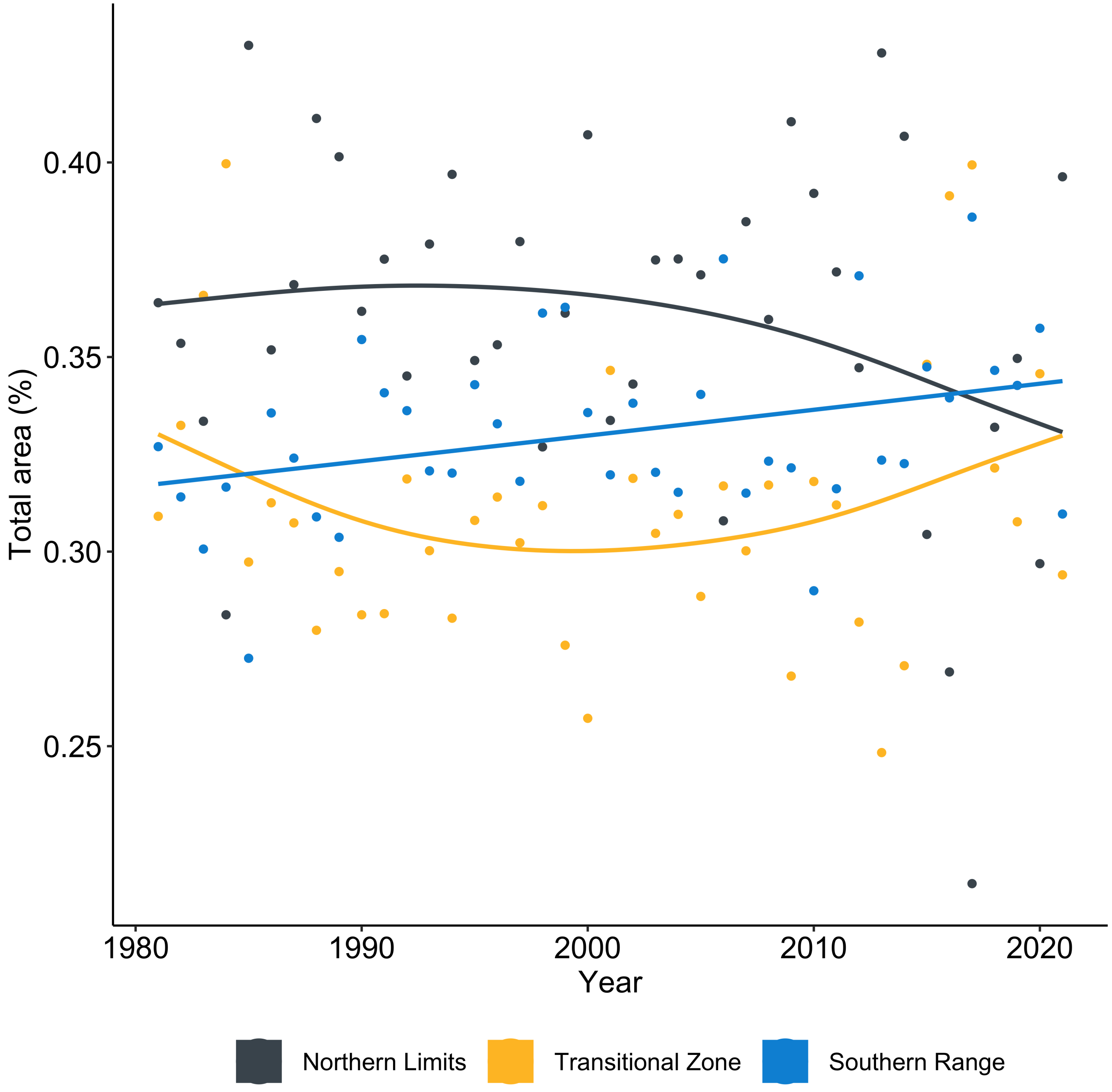

Fig. S1. Percent change in area for all zones between 1981 and 2021 for the map extent showing in Figure 4. Overall, there has been a consistent increase in Southern Range area. Transitional zone started increasing while the Northern Limits started decreasing in area after 2000. Trend lines are from a generalized additive model (Family: Beta, link: log) with an adjusted R^2 of approximately 0.30. Null space penalization was used to test for the existence of non-linear relationships.

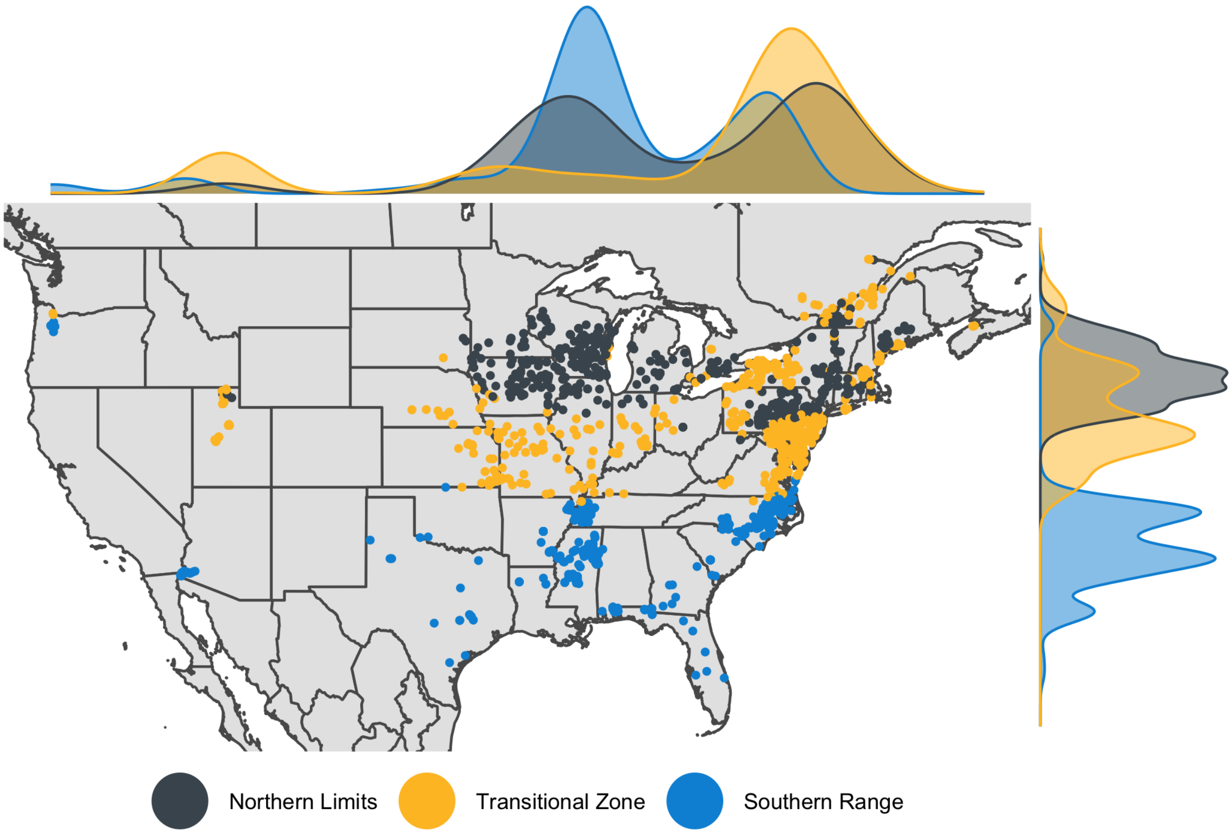

Fig. S2. Map of trap locations throughout the United States of America and Canada. Color represents the overwinter zone of the respective trap. Marginal graphs represent location densities for each zone. Traps are largely biased towards the Eastern Seaboard.

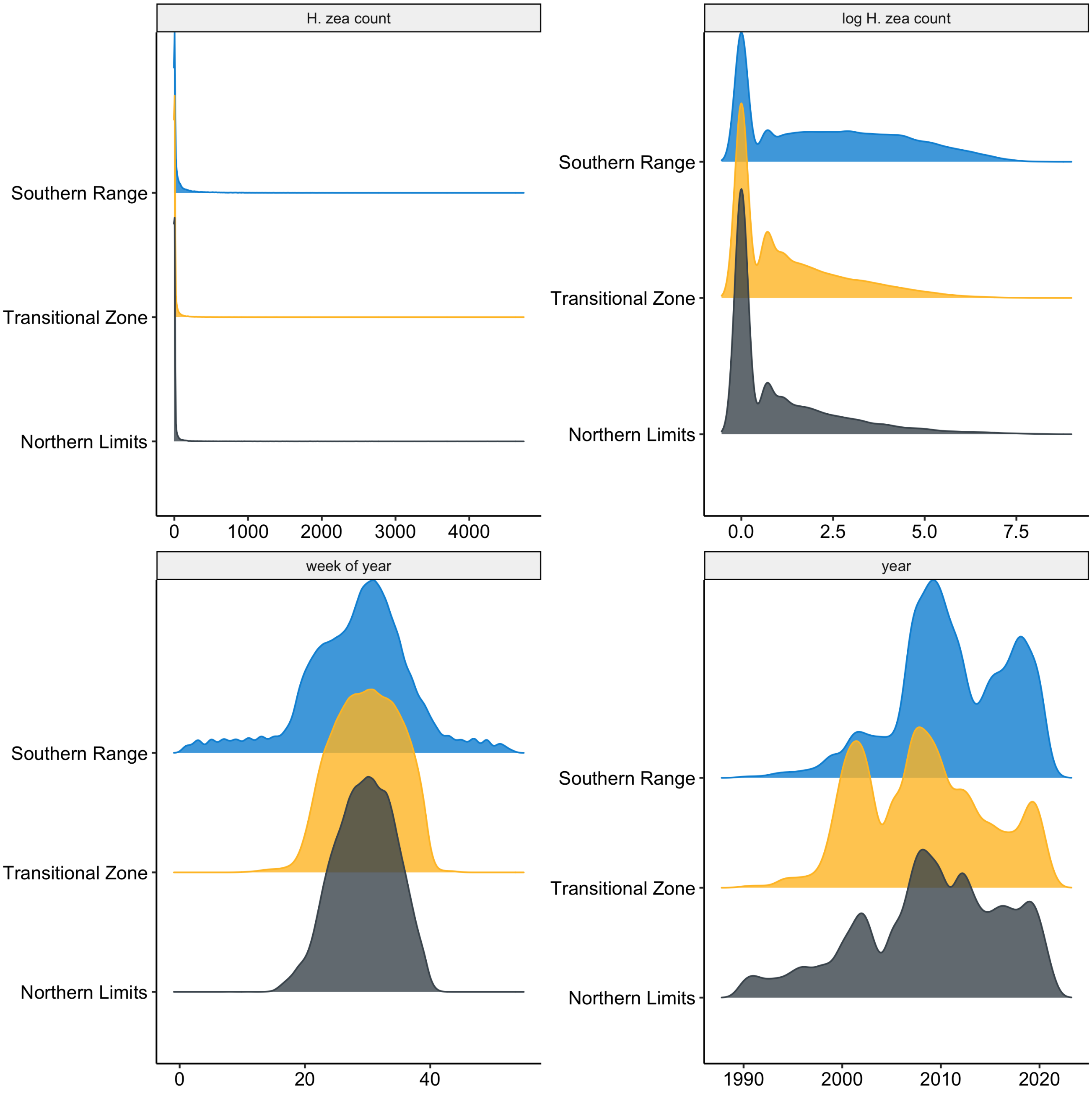

Fig. S3. Ridgeline plots showing the distribution of the data of *H. zea* counts (both raw and logged) and the distribution of observations through week of year and year broken into the three overwintering zones. Count data are heavily skewed with many zeros. Most trap observations happen during crop growing season which explains the distribution of week of year for all zones being between week of year 20 and 40. Trapping effort has increased over the years although there is considerable variation between and within zones.

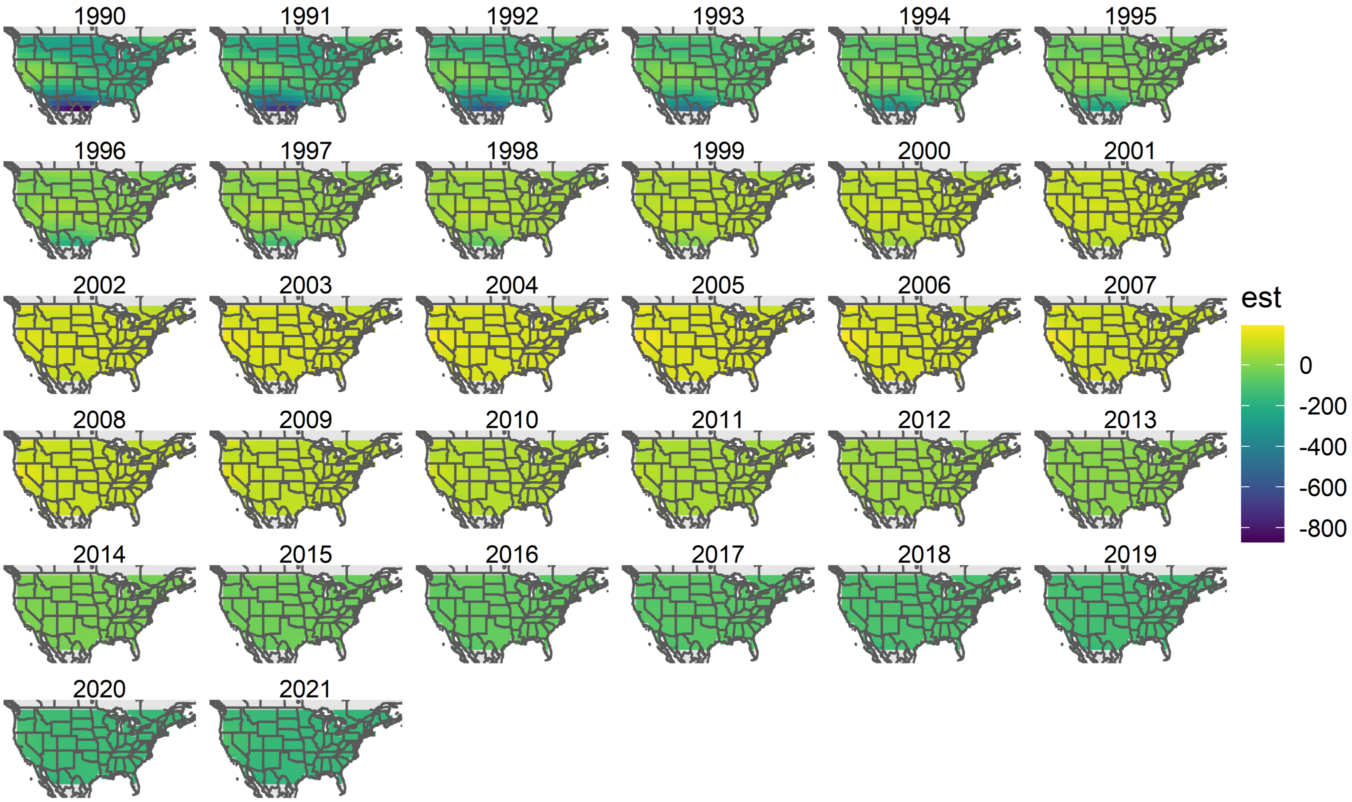

Fig. S4. The space-time tensor for latitude, longitude, and year. All models included this term. Colors represent the contribution of space and time (year) to *H. zea* count. Colors range from blue (negative relationship, lower *H. zea* counts) to yellow (positive, high *H. zea* count) model estimates. X and Y axes show longitude and latitude respectively while each facet represents a time slice from 1990 to 2021.

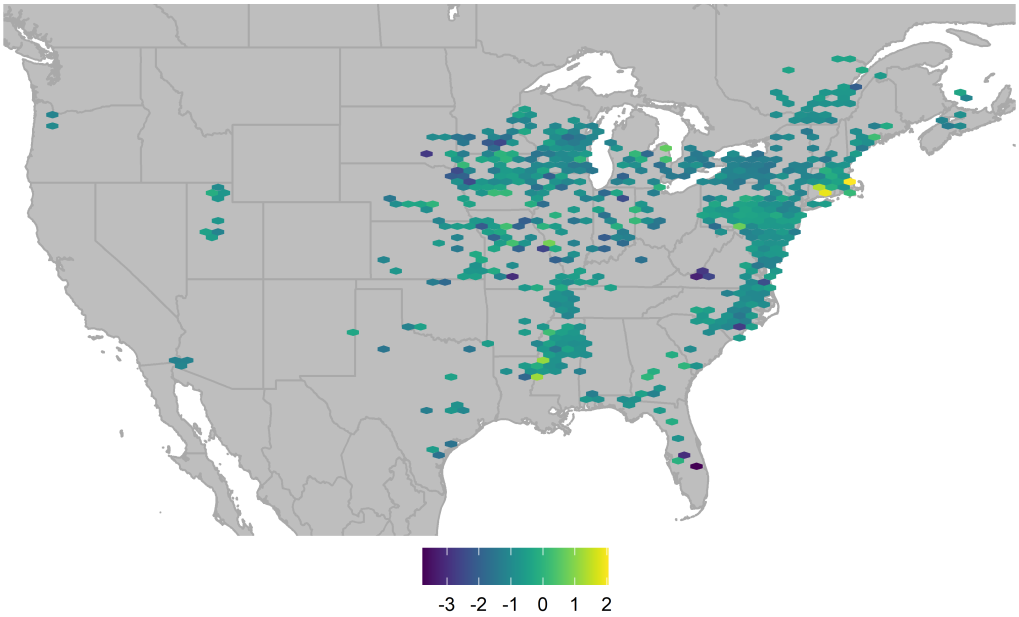

Fig. S5. Spatial correlation of remaining model GS residuals. Model description can be seen in the main document's statistical analysis section. Color represents the averaged residual value for that area. Areas that are brighter or darker indicate residual hotspots that are not explain by the model.

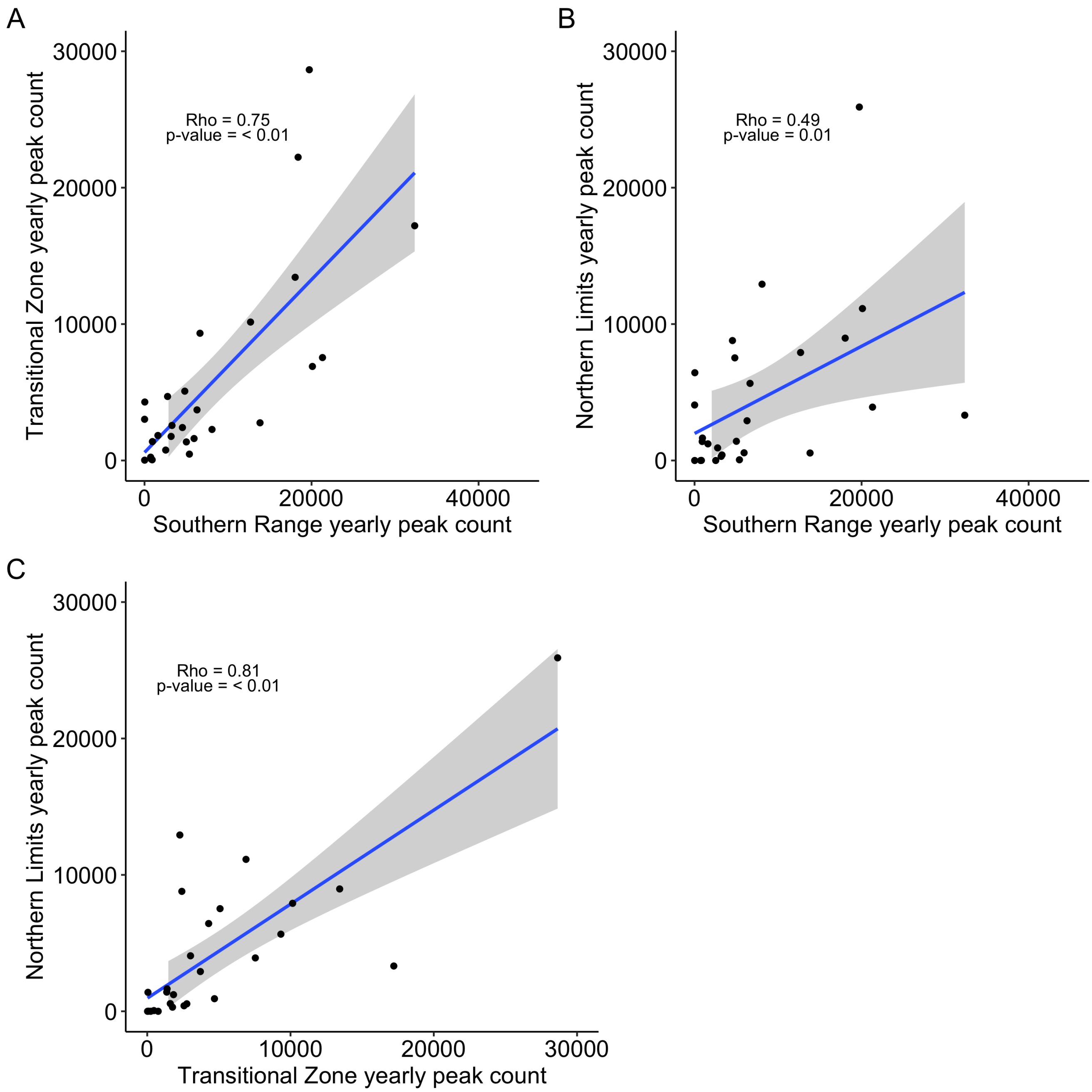

Fig. S6. Correlations of yearly *H. zea* counts between Transitional Zone and Southern Range (A), Northern Limits and Southern Range (B), and Northern Limits and Transitional Zone (C). All regions are strongly positively correlated between each other.

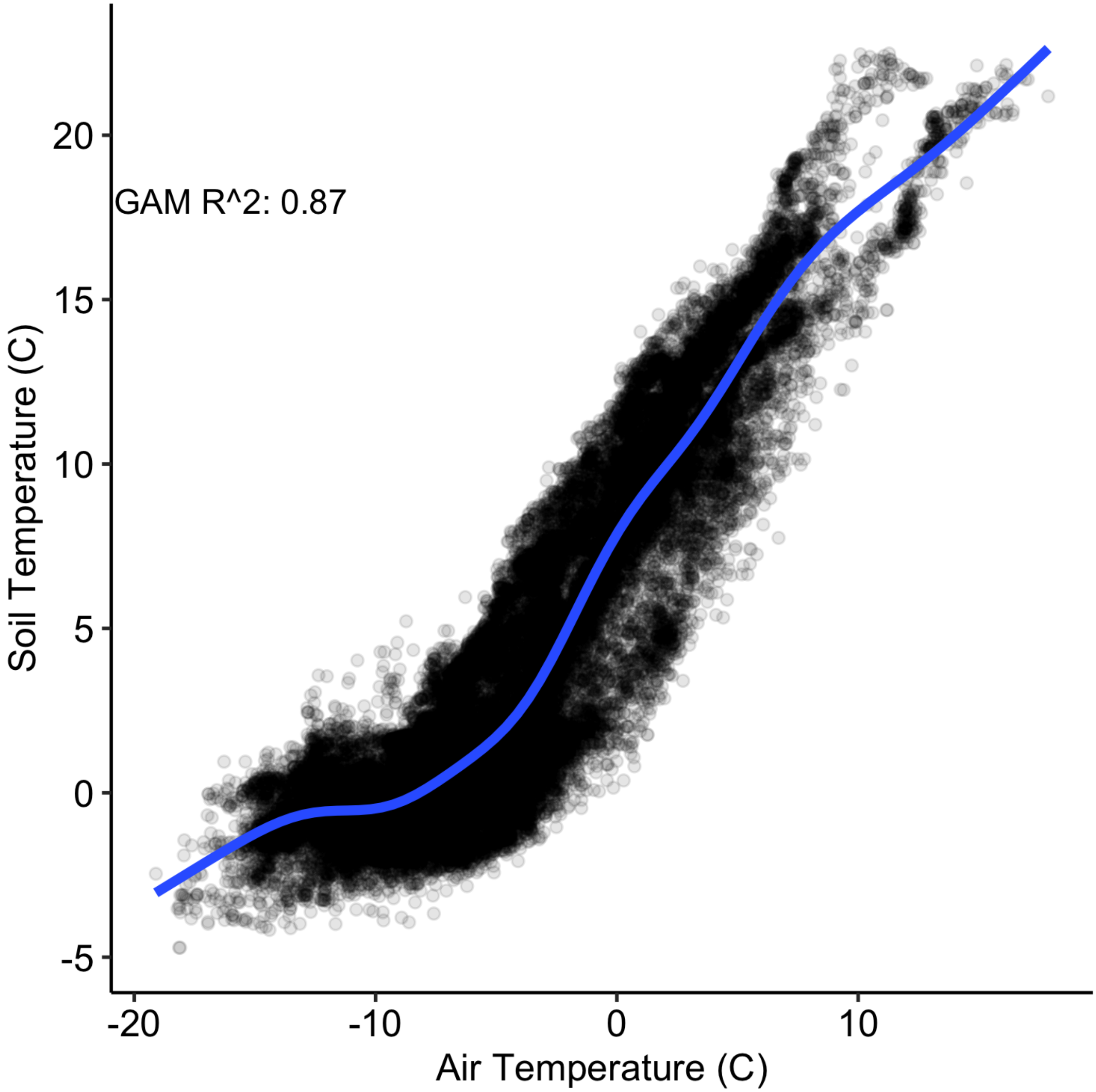

Fig. S7. Correlation between historic air and soil temperature used to predict future soil temperatures. Air temperatures were retrieved from NEX-DCP30 while the soil temperatures (up to 28 cm in depth) were retrieved from the ECMWF Climate Reanalysis databases. Line represents best fit from a generalized additive model (family: Gaussian, link: identity) with an adjusted r-square of 0.87.

Table S1. Structure testing model selection results. Lower AIC and BIC values indicate more support for each model. AIC = Akaike Information Criterion, BIC = Bayesian Information Criterion.

Model	BIC	deltaBIC	AIC	deltaAIC
40-year average three zone	581840	0	564377	0
40-year average two zone	581864	24	564409	31
year to year two zone	581912	72	564474	96
year to year three zone	581921	81	564472	95
no structure	581948	108	564519	141
40th parallel	581954	114	564514	136

Table S2. Model GS results. woy: week of year, EDF: estimated degrees freedom, DF: degrees freedom. This model has a global smoother with similarly smoother group-level trends

	Model GS			
variables	EDF	DF	F	p-value
s(woy)	5.18	13.00	1.79	0.00
s(year)	12.73	13.00	604.27	0.00
te(woy,overwinter zone)	23.38	38.00	781.00	0.00
te(year,overwinter zone)	42.17	45.00	477.02	0.00
te(Latitude,Longitude,year)	103.95	120.00	23606.69	0.00
s(location)	1627.94	1973.00	18.14	0.00
s(zone_30y)	0.00	3.00	0.00	0.00
s(trap type)	1.97	2.00	15137.99	0.00
deviance explained	0.70			
adjusted R^2	0.30			

Table S3. Model selection criteria for all models. Model descriptions can be found in the materials and methods section. BIC and AIC were derived from models that used all the data. OOS, RMSE, and R^2 are the mean results from a 10-fold cross validation. Delta columns represent the change from the smallest value (BIC, AIC, OOS, RMSE) or largest value (R^2). AIC = Akaike Information Criterion, BIC = Bayesian Information Criterion, OOS: Out of Sample Deviance, RMSE = out of sample Root Mean Square Error.

Models	OOS	delta OOS	RMSE	delta RMSE	R^2	delta R^2	AIC	delta AIC	BIC	delta BIC
GS	160894.66	0.00	127.30	0.78	0.30	0.17	545697.96	0.00	562947.30	0.00
GI	162475.29	1580.63	127.04	0.52	0.30	0.17	545777.12	79.16	562985.22	37.92
S	161475.81	581.15	126.52	0.00	0.29	0.16	547080.74	1382.78	564244.35	1297.05
I	165594.12	4699.46	128.38	1.86	0.29	0.16	547109.16	1411.20	564254.01	1306.71
G	173367.25	12472.60	143.97	17.46	0.14	0.01	555258.22	9560.26	572119.78	9172.48
N	174599.50	13704.84	140.19	16.54	0.13	0.00	555384.91	9686.95	572204.56	9257.26

Table S4. Out of Sample deviance (OOS) of all models broken into the three overwintering zones. Increased deviance indicates more uncertain predictions to the out-of-sample dataset. These values came from a 10-fold cross validation. NL: Northern Limits, TZ: Transitional Zone, SR: Southern Range.

Model	NL	NL delta OOS	TZ	TZ delta OOS	SR	SR delta OOS
Model GS	40129.58	0.00	62239.13	0.30	58525.95	1143.94
Model S	41102.32	972.74	62991.48	752.65	57382.01	0.00
Model GI	41654.07	1524.49	62238.83	0.00	58582.38	1200.37
Model I	44229.39	4099.81	63745.17	1506.34	57619.55	237.54
Model G	45758.83	5629.25	65266.07	3027.24	62342.35	4960.34
Model N	46019.40	5889.82	66108.10	3869.27	62472.02	5090.01

Table S5. Model S results for *H. zea*. EDF = estimated degrees freedom and woy = week of year.

	Model S			
variables	EDF	DF	F	p-value
te(woy,overwinter zone)	30.32	38.00	5011.76	0.00
te(year,overwinter zone)	37.00	44.00	603.86	0.00
te(Latitude,Longitude,year)	103.44	121.00	24844.40	0.00
s(location)	1636.55	1973.00	18.22	0.00
s(zone_30y)	0.00	3.00	0.00	0.00
s(trap type)	1.97	2.00	16001.57	0.00
deviance explained	0.70			
adjusted R^2	0.30			

Table S6. Model GI and I results for *H. zea*. EDF = estimated degrees freedom and woy = week of year.

	Model GI				Model I			
variables	EDF	DF	F	p-value	EDF	DF	F	p-value
s(woy)	0.45	13	0.04	0.00				
s(year)	12.88	13	610.04	0.00				
te(woy):northern limits	8.16	12	71.16	0.00	8.23	11.00	8231.66	0.00
te(woy):transitional zone	9.90	13	53.12	0.00	9.95	13.00	5982.44	0.00
te(woy):southern ranges	11.60	13	18.29	0.00	11.98	13.00	3325.91	0.00
te(year):northern limits	12.67	14	693.69	0.00	12.94	14.00	362.11	0.00
te(year):transitional zone	13.51	14	595.41	0.00	9.32	14.00	657.92	0.00
te(year):southern ranges	12.49	14	645.16	0.00	12.78	14.00	706.11	0.00
te(Latitude,Longitude,year)	101.71	119	23638.43	0.00	103.22	121.00	24769.29	0.00
s(location)	1627.65	1973	18.18	0.00	1636.18	1973.00	18.22	0.00
s(overwintering zones)	0.00	2	0.00	0.00	0.00	2.00	0.00	0.00
s(trap type)	1.97	2	16742.20	0.00	1.97	2.00	16937.62	0.00
deviance explained	0.70				0.70			
adjusted R^2	0.30				0.30			

Table S7. Model G and N results for *H. zea*. EDF = estimated degrees freedom and woy = week of year.

	Model G				Model N			
variables	EDF	DF	F	p-value	EDF	DF	F	p-value
s(woy)	12.40	13.00	10923.12	0.00	12.40	13.00	10623.94	0.00
s(year)	10.86	13.00	844.99	0.00	10.85	13.00	814.87	0.00
te(Latitude,Longitude,year)	106.17	120.00	87645.80	0.00	106.40	119.00	60092.15	0.00
s(location)	1644.35	1973.00	18.22	0.00	1641.67	1973.00	18.31	0.00
s(overwintering zone)	1.92	2.00	46722.29	0.00				
s(trap type)	1.98	2.00	28511.54	0.00	1.98	2.00	26768.53	0.00
deviance explained	0.66				0.66			
adjusted R^2	0.13				0.13			

Table S8. Basis dimensions check for model GS and model S. EDF = estimated degrees freedom, k' = knot, and woy = week of year.

	Model GS				Model S			
Model terms	k'	EDF	k-index	p-value	k'	EDF	k-index	p-value
s(woy)	13	5.17	0.83	0.74				
s(year)	13	12.73	0.82	0.32				
te(woy,overwintering zone)	42	23.39	NA	NA	42.00	30.32	NA	NA
te(year,overwintering zone)	45	42.14	NA	NA	45.00	37.00	NA	NA
te(Latitude,Longitude,year)	124	104.27	0.75	0	124.00	103.44	0.75	0.00
s(location)	1974	1636.91	NA	NA	1974.00	1636.55	NA	NA
s(overwintering zone)	3	0	NA	NA	3.00	0.00	NA	NA
s(trap_type)	3	1.9715	NA	NA	3.00	1.97	NA	NA

Table S9. Basis dimensions check for model GI and model I. EDF = estimated degrees freedom, k’ = knot, and woy = week of year.

	Model GI				Model I			
Model terms	k’	EDF	k-index	p-value	k’	EDF	k-index	p-value
s(woy)	13.00	0.45	0.80	0.09				
s(year)	13.00	12.88	0.83	0.87				
te(woy):Northern Limits	13.00	8.16	0.80	0.08	13.00	8.23	0.82	0.26
te(woy):Transitional Zone	13.00	9.90	0.80	0.08	13.00	9.95	0.82	0.24
te(woy):Southern Ranges	13.00	11.60	0.80	0.11	13.00	11.98	0.82	0.24
te(year):Northern Limits	14.00	12.67	0.83	0.90	14.00	12.94	0.81	0.12
te(year):Transitional Zone	14.00	13.51	0.83	0.89	14.00	9.32	0.81	0.13
te(year):Southern Ranges	14.00	12.49	0.83	0.88	14.00	12.78	0.81	0.11
te(Latitude,Longitude,year)	124.00	101.71	0.74	0.00	124.00	103.22	0.76	0.00
s(location)	1974.00	1627.65	NA	NA	1974.00	1636.18	NA	NA
s(overwintering zone)	3.00	0.00	NA	NA	3.00	0.00	NA	NA
s(trap_type)	3.00	1.97	NA	NA	3.00	1.97	NA	NA

Table S10. Basis dimensions check for model G and model N. EDF = estimated degrees freedom, k' = knot, and woy = week of year.

	Model G				Model N			
Model terms	k'	EDF	k-index	p-value	k'	EDF	k-index	p-value
s(woy)	13.00	12.40	0.79	0.13	13.00	12.40	0.81	0.20
s(year)	13.00	10.86	0.80	0.29	13.00	10.85	0.80	0.07
te(Latitude,Longitude,year)	124.00	106.17	0.72	0.00	124.00	106.40	0.75	0.00
s(location)	1974.00	1644.35	NA	NA	1974.00	1641.67	NA	NA
s(overwintering zone)	3.00	1.92	NA	NA				
s(trap_type)	3.00	1.98	NA	NA	3.00	1.98	NA	NA

Movie S1. Change in overwintering zone area overtime from 1981 to 2021. As can be seen, there is considerable spatial and temporal variability around the zones with the transitional zone being the most variable.

References

1. MCK Tweedie, An index which distinguishes between some important exponential families in *Statistics: Applications and new directions: Proc. Indian statistical institute golden Jubilee International conference*. Vol. 579, pp. 579–604 (1984).
2. SN Wood, *Generalized additive models: an introduction with R*. (Chapman and Hall/CRC), (2017).
3. R Core Team, R: A language and environment for statistical computing (2022).
4. H Wickham, Tidyverse: easily install and load the 'Tidyverse'. 2017. R package version 1.2. 1 (2018).
5. N Gorelick, et al., Google Earth Engine: Planetary-scale geospatial analysis for everyone. *Remote. sensing Environ.* **202**, 18–27 (2017).
6. GL Simpson, *gratia: Graceful ggplot-Based Graphics and Other Functions for GAMs Fitted using mgcv*, (2022) R package version 0.7.0.
7. G Grolemund, H Wickham, Dates and times made easy with lubridate. *J. Stat. Softw.* **40**, 1–25 (2011).
8. E Pebesma, RS Bivand, S classes and methods for spatial data: the sp package. *R news* **5**, 9–13 (2005).
9. E Pebesma, Simple Features for R: Standardized Support for Spatial Vector Data. *The R J.* **10**, 439–446 (2018).
10. RJ Hijmans, J van Etten, Raster: geographic data analysis and modeling. R package (2015).
11. A South, Rnaturalearth: world map data from natural earth. *R package version 0.1. 0* (2017).
12. R Bivand, T Keitt, B Rowlingson, rgdal: Bindings for the 'Geospatial' Data Abstraction Library. (2020).
13. JS Evans, spatialeco (2021) R package version 1.3-6.
